# Prevalence-pattern and risk factors of Cesarean section in a multiethnic cohort

**DOI:** 10.12669/pjms.37.3.3186

**Published:** 2021

**Authors:** Khadija Murtaza, Madeeha Chaudhry, Shabana Nazeer, Sajid Malik

**Affiliations:** 1 Khadija Murtaza, Human Genetics Program, Department of Zoology, Faculty of Biological Sciences, Quaid-i-Azam University, Islamabad 45320, Pakistan; 2 Madeeha Chaudhry, Human Genetics Program, Department of Zoology, Faculty of Biological Sciences, Quaid-i-Azam University, Islamabad 45320, Pakistan; 3 Shabana Nazeer, Human Genetics Program, Department of Zoology, Faculty of Biological Sciences, Quaid-i-Azam University, Islamabad 45320, Pakistan; 4 Sajid Malik, Human Genetics Program, Department of Zoology, Faculty of Biological Sciences, Quaid-i-Azam University, Islamabad 45320, Pakistan

**Keywords:** parturition, birth, maternal health, mode of delivery, obstetric delivery, pregnancy complications

## Abstract

**Objective:**

This study was aimed to elucidate the prevalence-pattern and determinant of cesarean section (CS) in a multiethnic cohort from Pakistan.

**Methods:**

Through a cross-sectional study design, women delivering at a tertiary care center were recruited during 2013-2017. Data on socio-demographic variables, obstetric complications and birth outcome were obtained. Descriptive statistics, bivariate and multivariate logistic regression analyses were performed.

**Results:**

A total of 5,275 pregnant women were recruited and 43% of the deliveries underwent CS. Odds of CS were significantly higher in subjects originating from Azad JammuKashmir and Sindh regions, speaking Potohari and Pahari languages, women in advance ages, and those who were housewives. CS had significantly lower odds of prenatal mortality but increased odds of postnatal mortality. Obstetric factors that appeared to be significant predictors of CS were multiparity, breech position, fetal distress, oligohydroamniosis, preeclampsia, and previous scar.

**Conclusion:**

This study revealed high variability in CS in various socio-demographic strata of study population. The obstetric complications highlighted in this study may be reduced by proper perinatal counseling and pregnancy monitoring and should be the focus of intervention programs as suggested in the Millennium Development Goals.

## INTRODUCTION

In a woman’s life span, pregnancy is a critical period being recognized as welcome event for successful womanhood,^1^ while delivery is one of the most crucial period of pregnancy culminating in birth of baby. Modes of delivery can be either the natural (vaginal) or by surgical intervention (Cesarean section; CS). The rate of Cesarean section (CSR) is continuously rising in many countries around the globe and has exceeded the level of 10–15% which is recommended by World Health Organization.^2^,^3^ Unnecessary CS may be associated with an increased risk of maternal and neonatal mortality, long-term health of child and mothers as well as future pregnancies.^3^,^4^

Pakistan is the 5^th^ largest contributor to worldwide maternal mortality and an overall 6% of the world’s maternal deaths occur here. One of the important objectives of Millennium Development Goals (MDGs) is to reduce maternal and child mortality which may be addressed by understanding the determinants of maternal and obstetric well-being.^5^ In resource deficient countries with inadequate access to health facilities, it is vital to prioritize areas of interventions and vulnerable patient population.^4^ To this end, this study was carried out with the aim to elucidate the prevalence-pattern and risk factors associated with CS in a multiethnic Pakistani cohort.

## METHODS

This study was carried out at the Mother-Child Health Centre (MCH), Pakistan Institute of Medical Sciences (PIMS), Islamabad. The study was approved by ethical committee of (Ref: DAS/13-HG-6, Dated: June3, 2013) Quaid-i-Azam University and PIMS.

The study subjects were pregnant women delivering at MCH during 2013-2017. Only the current pregnancy was included in the analyses. All deliveries, i.e., vaginal and CS, either term/preterm/postdated, singleton/multiple, and nulliparous/multiparous, were included. Data of women giving incomplete information and few of the highly risky or complicated pregnancies brought to the critical-care unit were not included. Data regarding socio-demographic variables, obstetric complications, birth parameters and perinatal outcomes were obtained on a structured proforma.

### Statistical analyses:

Descriptive statistics involving frequency and percentages was employed to characterize the sample. Bivariate and multivariate logistic regression analyses were used to ascertain the association between dependent variable (CS) and independent variables (STATA, ver.11). Multivariate analyses were performed by employing all variables in a step-by-step approach and only the significant variables were retained in the final model. Results were expressed by crude odd ratio (COR) and adjusted odd ratio (AOR).

## RESULTS

### Sample characteristics:

A total of 5,275 women were recruited (mean age 28.8±5.1 years). There was highest representation of participants from twin cities Islamabad/Rawalpindi (46%), urban origin (63%), speaking Punjabi language (62%), literate (79%), housewives (96%), and belonging to middle economic quintile (42%) and joint family system (58%).

### Socio-demographic differentials of CS:

CS was observed in 43% of the deliveries and normal deliveries were 57%. There was high variation in CSR in various socio-demographic attributes of the subjects.

Multivariable logistic regression analyses revealed that four out of eight socio-demographic variables, i.e., province, mother tongue, age category and women’s occupational status were significant predictors of CS (Table-I). Women originating from Azad Jammu-Kashmir (AJK) (AOR:1.33; 95% CI:0.97–1.81) and Sindh province (AOR:1.32; 95% CI:0.88-1.97) were more likely to deliver children by CS. Likewise, women speaking Potohari (AOR:1.47; 95% CI: 1.03-2.10), Urdu (AOR:1.35; 95% CI: 0.96-1.92), Pahari (AOR:1.34; 95% CI: 0.99-1.83) and Saraiki (AOR:1.34; 95% CI: 0.85-2.11) languages were more likely to have CS. The women with advanced age (AOR: 2.25; 95% CI: 1.85-2.75) had the higher risk of delivering children by CS. Further, women who were housewives (AOR:1.58; 95% CI:1.19-2.08) had higher risk of CS compared to women engaged in certain professions. The variables like rural/urban residence, subject education, economic quintile and household type were statistically not significant (data not shown).

**Table-I:** Socio-demographic differentials in birth type and risk of CS.

Variable*	Normal birth (%)	Cesarean section (%)	Total (n)	Adjusted OR	95% CI	P-value
***Origin (province)***						
Khyber Pakhtunkhwa	60	40	847	Ref.		
Islamabad/Rawalpindi	57	43	2,415	1.00	0.80-1.25	0.997
Punjab (Upper)	56	44	1,111	1.02	0.80-1.30	0.892
Azad Jammu Kashmir	49	51	388	1.33	0.97-1.81	0.073
Punjab (Southern)	60	40	330	0.84	0.61-1.16	0.281
Sindh	51	49	127	1.32	0.88-1.97	0.177
Others	64	36	57	0.88	0.47-1.65	0.681
**Total**	**57**	**43**	**5,275**			
***Mother tongue***						
Pushto	63	37	619	Ref.		
Punjabi	56	44	3,271	1.33	1.05-1.69	0.018
Pahari	54	46	403	1.34	0.99-1.83	0.062
Potohari	52	48	222	1.47	1.03-2.10	0.032
Urdu	55	45	218	1.35	0.96-1.92	0.088
Hindko	56	44	215	1.30	0.95-1.80	0.105
Saraiki	60	40	129	1.34	0.85-2.11	0.214
Kashmiri	56	44	95	1.08	0.66-1.79	0.751
Others	59	41	103	1.16	0.72-1.87	0.537
***Age category (yrs)***						
19-24	66	34	1,720	Ref.		
25-34	53	47	3,004	1.67	1.48-1.89	<0.0001
>34	47	53	551	2.25	1.85-2.75	<0.0001
***Mother occupation***						
Working women	64	36	233	Ref.		
Housewife	56	44	5,000	1.58	1.19-2.08	0.001
_cons				0.26	0.18-0.36	<0.0001

Ref.=Reference category;

* only significant variables have been reported

### Birth outcome, pregnancy and delivery related predictors of CS:

The likelihood of CS was 8% higher in pregnancies bearing daughters as compared to sons (COR:1.08; 95% CI:0.96-1.21) (Table-II). CS had significantly lower odds in prenatal mortality (COR:0.37; 95% CI: 0.26-0.52) but increased likelihood of postnatal mortality (COR: 2.12; 95% CI: 1.09-4.14). The odds of CS were 23% higher in multiparous women as compared to nulliparous women (COR:1.23; 95% CI:1.09-1.38). Further, women with previous history of fetal/prenatal loss had 20% higher risk of CS (COR:1.20; 95% CI: 1.06-1.36) (Table-II).

**Table-II:** Birth outcome and pregnancy and delivery related predictors of CS.

Variable	Normal delivery (%)	Cesarean section (%)	Total (n)	Crude OR	95% CI	P-value
***Birth outcome/history***						
Gender of neonate						
Son	57	43	2,509	Ref.		
Daughter	55	45	2,391	1.08	0.96-1.21	0.020
Total	57	43	4,900			
***Pregnancy outcome***						
Alive	56	44	4,900	Ref.		
Prenatal mortality	78	22	189	0.37	0.26-0.52	<0.0001
Postnatal mortality	38	62	37	2.12	1.09-4.14	0.032
***Parity***						
Nulliparous	60	40	1,714	Ref.		
Multiparous	55	45	3,565	1.23	1.09-1.38	<0.0001
***History of fetal/prenatal loss***						
No	58	42	3,769	Ref.		
Yes	54	46	1,357	1.20	1.06-1.36	0.013

*Obstetric complication#*				*Adjusted OR*	*95% CI*	*P-value*

**Multiple pregnancy**						
Singleton	57	43	5,125	Omitted		
Multiple pregnancy	42	58	154			
Breech*	17	83	258	8.59	5.98-12.33	<0.0001
Fetal distress*	7	93	207	25.26	13.57-47.01	<0.0001
Oligohydroamniosis*	31	69	331	2.26	1.71-3.00	<0.0001
Preeclampsia*	32	68	129	2.70	1.79-4.09	<0.0001
Previous scar*	10	90	321	16.32	11.01-24.20	<0.0001
Duration of pregnancy (>37 weeks)*	57	43	2,233	0.72	0.63-0.81	<0.0001
Bleeding	60	40	112	0.89	0.61-1.30	0.554
_cons				0.58	0.53-0.63	<0.0001

Ref.=Reference category; #=included in multivariable logistic regression,

* =significant in multivariable logistic regression

There was a higher risk of CS multiple pregnancy as compared to singleton (58% vs. 43, respectively; COR:1.82; 95% CI: 1.31-2.52). In order to assess the predictors of CS, multivariable logistic regression analyses were performed in two tires. In the first round, four variables were analyzed, i.e., gender of neonate, pregnancy outcome, parity and history of fetal/prenatal loss. In these analyses, no variable appeared significant. In the second round, the remaining factors were taken simultaneously. Here, seven variables emerged significant (Table-II). The highest predictors of CS were fetal distress (AOR:25.26; 95% CI:13.57-47.01), followed by previous scar (AOR:16.32; 95% CI: 11.01-24.20) and breech (AOR: 8.59; 95% CI:5.98-12.33). Interestingly, duration of pregnancy (>37 weeks) also emerged as significant predictor (AOR:0.72; 95% CI: 0.63-0.81).

## DISCUSSION

It is first detailed study from Pakistan reporting a large number of socio-demographic, obstetric and birth outcome variables associated with CSR. This study showed that the prevalence of CS was 43%. A wide variability has been witnessed in CSR in various cultures. For instance, 50% in Iran,^6^ and 28% in Brazil.^7^ In Pakistan, CSR was reported to range from 47% in Abbottabad to 65% in Hyderabad.^8^,^9^ Differentials in the sociocultural and economic factors and available resources may account for this variability.

Multivariable analyses revealed that four socio-demographic variables were significantly associated with CS, i.e., province, mother tongue, age category and occupational status were significant predictors of CS. Here, the women from AJK and Sindh had the highest prevalence of CS. Previously, Manzoor et al. also reported a high rate of CS in AJK. Further, the highest prevalence of CS is in Potohari speaking subjects (48%), while lowest in Pushto speaking women.^10^ This is contrary to a prior Pakistani study by Mumtaz et al. in which the rate of CS was highest in Urdu speaking women (29%) followed by Punjabi speaking women (23%).^11^ The high CSR in subject from AJK, who mostly observed Potohari and Pahari languages, could be explained due to the fact that AJK region is deprived of advanced medical care facilities and the earthquake and other natural disasters in the recent past have heavily damaged the tertiary care hospitals. Hence, the women from the adjoining regions of AJK having pregnancy complications are inclined to visit MCH, PIMS.

The prevalence of CS was observed to be increasing with increasing maternal age. In another study carried out in Pakistan, the likelihood of caesarean deliveries was associated with mothers aged more than 24 years.^12^ In the Pakistani context, it has been argued that because of career, financial and educational orientation, there is a rising trend among women of getting married or conceiving at a later age which tends to increase their chances of undergoing CS.^13^ Furthermore, CSR was observed to be higher in housewives as compared to working women which is consistent with the observation of Mumtaz et al.^11^

Obstetric complications have been known to increase the risk of CS; however, the combination of obstetric factors detrimental to CS vary across populations. The multivariable analyses revealed that nearly all studied obstetric factors were contributing to the risk of CS. It is nonetheless, interesting to see the differential contribution of variables in the final model (Table-II). The highest risk was imposed by fetal distress, previous scar, and breech position. On the other hand, the duration of pregnancy (long) was observed in to reduce the risk of CS. Curiously however, this study showed no association of CS and bleeding. Bleeding may be an indication of serious pregnancy condition particularly in women with previous history of obstetric complications.

In addition to the socio-demographic and obstetric variables, potentially other confounding factors such as women choice, women fear, psychological factors, and doctors’ preference are also responsible for the higher CSR. As suggested in the MDGs, it is possible to minimize the CSR by adequate awareness, proper prenatal and perinatal counseling of the patients, monitoring of feto-maternal parameters, careful selection of the patient who have previous CS and promoting institutional deliveries.^14^ Thus, CS should be performed when medically necessary, ascertained by the healthcare providers caring for the woman on a case-by-case basis.^4^

### Limitations of the study:

Since, it is a hospital-based study and mostly the complicated cases that need CS visit here; therefore, such cases may be overrepresented in these data. In the rural areas of Pakistan, home deliveries by traditional birth attendants are a cultural norm. Hence, there may not be full representation of subjects from rural and remote areas in this sample.

## CONCLUSIONS

This study witnessed high variability in CSR in various Pakistani socio-demographic strata and helps identify the potential areas of intervention. For instance, women from AJK and Sindh should be provided proper perinatal counseling and pregnancy monitoring. The obstetric complications may the focus of intervention programs intended to increase women well-being and reducing the CSR.

### Authors Contribution:

**SM:** Conceived, designed and planned the study, performed statistical analyses and is responsible for the integrity of study.

**MC & SN:** Helped in data collection.

**KM:** Prepared initial draft; helped analyze the data.

**KM, MC, SN & SM:** Edited, reviewed and approved the manuscript.

## References

[1] Vijayalaxmi KG, Urooj A (2009). Influence of Maternal Factors on Mode of Delivery and Birth Weight in Urban Pregnant Women. J Hum Ecol.

[2] Betran AP, Torloni MR, Zhang JJ, Gulmezoglu AM (2016). WHO statement on caesarean section rates:a commentary. BJOG Obst Gynaecol.

[3] Betran AP, Merialdi M, Lauer JA, Bing-Shun W, Thomas J, Van Look P (2007). Rates of caesarean section:analysis of global, regional and national estimates. Paediatr Perinat Epidemiol.

[4] Gibbons L, Belizan J, Lauer J, Betran A, Merialdi M, Althabe F (2010). The Global numbers and costs of additionally needed and unnecessary caesarean sections performed per year:Overuse as a barrier to universal coverage. World Health Report.

[5] Hogan MC, Foreman KJ, Naghavi M, Ahn SY, Wang M, Makela SM (2010). Maternal mortality for 181 countries, 1980–2008:a systematic analysis of progress towards Millennium Development Goal 5. Lancet.

[6] Abdoli M, Amouzeshi Z, Nakhaee MH (2014). Prevalence of cesarean section and related causes of in women referring to Vali-e-Asr and Tamin-e-Ejtemaee hospitals in Birjand, 2010. J Surg Traum.

[7] Faisal-Cury A, Menezes PR, Quayle J, Santiago K, Matijasevich A (2017). The relationship between indicators of socioeconomic status and cesarean section in public hospitals. Rev Saude Publica.

[8] Tahir N, Adil M, Fatima S, Khan S (2018). Cesarian sections:Frequency and indications at peripheral tertiary care hospital. Pak Armed Forces Med J.

[9] Haider G, Zehra N, Munir AA, Haider A (2009). Frequency and indications of cesarean section in a tertiary care hospital. Pak J Med Sci.

[10] Manzoor K, Malik QA, Raja MS, Raja MI (2016). Trend of caesarean sections in Azad Jammu &Kashmir. Int J Patholo.

[11] Mumtaz S, Bahk J, Khang Y-H (2017). Rising trends and inequalities in cesarean section rates in Pakistan:Evidence from Pakistan Demographic and Health Surveys, 1990-2013. PLoS ONE.

[12] Amjad A, Amjad U, Zakar R, Usman A, Zakar MZ, Fischer F (2018). Factors associated with caesarean deliveries among child-bearing women in Pakistan:secondary analysis of data from the Demographic and Health Survey, 2012–13. BMC Preg Childbirth.

[13] Hanif HM (2011). Association between maternal age and pregnancy outcome:implications for the Pakistani society. J Pak Med Assoc.

[14] Gonda A, Bukhari S, Karim MT, Karim S (2017). Frequency of caesarean section at a tertiary care hospital. J Sheikh Zayed Med Coll.

